# CRISPR screen of venetoclax response-associated genes identifies transcription factor ZNF740 as a key functional regulator

**DOI:** 10.1038/s41419-024-06995-x

**Published:** 2024-08-27

**Authors:** Lixia Zhang, Xinyue Zhou, Sajesan Aryal, Virginia Veasey, Pengcheng Zhang, Fu Jun Li, Yu Luan, Ravi Bhatia, Yang Zhou, Rui Lu

**Affiliations:** 1 Division of Hematology/Oncology, Department of Medicine, University of Alabama at Birmingham Heersink School of Medicine, Birmingham, AL USA; 2 O’Neal Comprehensive Cancer Center, University of Alabama at Birmingham Heersink School of Medicine, Birmingham, AL USA; 3 Department of Hematology, The First Affiliated Hospital of Chongqing Medical University, Chongqing, China; 4 Department of Cell Systems and Anatomy, University of Texas Health San Antonio, San Antonio, TX USA; 5 Greehey Children’s Cancer Research Institute, University of Texas Health San Antonio, San Antonio, TX USA; 6 Department of Biomedical Engineering, School of Medicine and School of Engineering, University of Alabama at Birmingham, Birmingham, AL USA

**Keywords:** Acute myeloid leukaemia, Transcriptomics, Apoptosis

## Abstract

BCL-2 inhibitors such as venetoclax offer therapeutic promise in acute myeloid leukemia (AML) and other cancers, but drug resistance poses a significant challenge. It is crucial to understand the mechanisms that regulate venetoclax response. While correlative studies have identified numerous genes linked to venetoclax sensitivity, their direct impact on the drug response remains unclear. In this study, we targeted around 1400 genes upregulated in venetoclax-sensitive primary AML samples and carried out a CRISPR knockout screen to evaluate their direct effects on venetoclax response. Our screen identified the transcription factor ZNF740 as a critical regulator, with its expression consistently predicting venetoclax sensitivity across subtypes of the FAB classification. ZNF740 depletion leads to increased resistance to ventoclax, while its overexpression enhances sensitivity to the drug. Mechanistically, our integrative transcriptomic and genomic analysis identifies NOXA as a direct target of ZNF740, which negatively regulates MCL-1 protein stability. Loss of ZNF740 downregulates NOXA and increases the steady state protein levels of MCL-1 in AML cells. Restoring NOXA expression in ZNF740-depleted cells re-sensitizes AML cells to venetoclax treatment. Furthermore, we demonstrated that dual targeting of MCL-1 and BCL-2 effectively treats ZNF740-deficient AML in vivo. Together, our work systematically elucidates the causal relationship between venetoclax response signature genes and establishes ZNF740 as a novel transcription factor regulating venetoclax sensitivity.

## Introduction

Acute myeloid leukemia (AML) is a hematopoietic malignancy marked by abnormal cell proliferation, hindered differentiation, and disrupted apoptosis. Despite treatment advancements, the prognosis remains poor, with less than 30% of adult AML patients surviving over five years [1]. AML cells often utilize anti-apoptotic mechanisms to resist therapy-induced apoptosis. The introduction of the BCL-2 inhibitor venetoclax, especially in combination with hypomethylating agents, has been a notable development in AML treatment [2, 3]. Yet, the challenge of drug resistance significantly impacts its effectiveness, highlighting the critical need to unravel the mechanisms underlying venetoclax efficacy in AML.

The mechanisms of resistance to venetoclax in AML involve various genetic, cellular, and metabolic processes [4]. Genetic aberrations such as TP53 mutations, FLT3 kinase activation, and other survival kinase signaling pathways, along with acquired loss-of-function mutations in key apoptotic regulators like BAX, contribute to adaptive resistance to venetoclax-based therapies [5–9]. Additionally, overexpression of anti-apoptotic proteins such as MCL-1, BCL-xL, and BCL2A1, which venetoclax does not target, can negate the effects of BCL-2 inhibition, leading to reduced drug efficacy [7, 10]. Recent studies also suggest mitochondrial adaptations, cellular lineage bias, and alterations in metabolic states as contributing factors to venetoclax resistance mechanisms [11–15]. Despite these insights, the transcriptional mechanisms regulating venetoclax response are less understood. In addition, while transcriptome analyses in primary samples have identified numerous genes linked to venetoclax sensitivity [16], it remains crucial to distinguish between genes that play a direct role and those that are simply associated.

In this study, we conducted CRISPR knockout screens to interrogate the functional roles of genes associated with clinical venetoclax sensitivity. By integrating genetic, pharmacological, transcriptomic, and genomic approaches, we identified the transcription factor ZNF740 as a crucial regulator of venetoclax sensitivity and uncover a potential ZNF740-NOXA-MCL-1 axis that governs the cellular response to BCL-2 inhibition in AML. Furthermore, our findings demonstrate that combined inhibition of MCL-1 and BCL-2 may be used to effectively treat AMLs with diminished ZNF740 expression.

## Results

### CRISPR screen of venetoclax response-associated genes reveals transcription factor ZNF740 as a key regulator

**Fig. 1:**
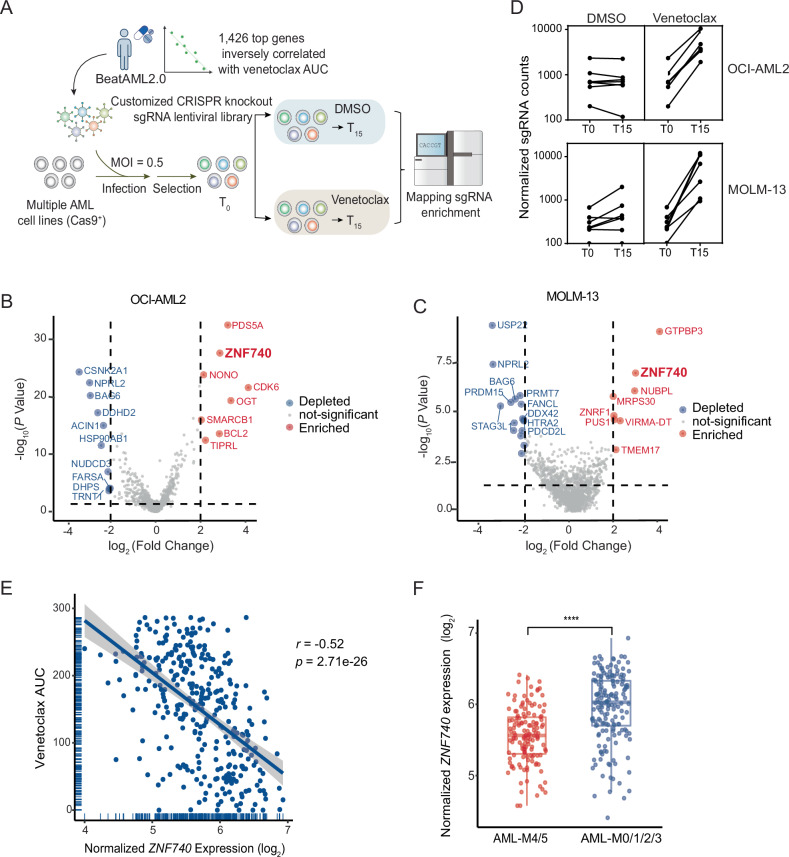
CRISPR screen of venetoclax response-associated genes reveals transcription factor ZNF740 as a key regulator. **A** Schematic overview of the CRISPR knockout screen. OCI-AML2 and MOLM-13 cells with stable Cas9 expression were transduced with pooled sgRNAs in lentiviruses. After antibiotic selection, these cells were treated with DMSO or venetoclax for 15 days. Following treatment, genomic DNA was harvested for library construction and analyzed through next-generation sequencing. **B**, **C** Volcano plots showing the log_2_ fold change of normalized sgRNA counts and −log_10_ *p* values from the screen results in OCI-AML2 and MOLM-13 cell lines. **D** Line plots depicting the normalized counts of ZNF740 sgRNAs over time, under treatments of either DMSO or venetoclax. **E** Scatter plot representing the negative correlation between normalized ZNF740 expression level and venetoclax AUC in AML patient samples of all FAB subtypes from BeatAML2.0. **F** Bar plot showing normalized ZNF740 expression levels in AML-M4/5 and other FAB subtypes in AML patient samples from the BeatAML2.0 database. ****p < 0.0001.

Previous studies in the BeatAML 2.0 project have pinpointed thousands of genes correlated with up-front venetoclax response [16], yet the causal role of these genes in influencing venetoclax sensitivity remains unexplored. Here, we focused on 367 BeatAML samples for which both gene expression and venetoclax response data were available. The majority of these samples (248/367, 67.6%) were from initial diagnoses, while the remaining samples were from cases of relapse, remission, or residual AML (Supplementary Fig. 1A). We identified 1426 top genes whose expression inversely correlated with venetoclax AUCs (area under the curve, Pearson correlation *r* < −0.40) in these primary AML samples (Supplementary Fig. 1B). We hypothesized that if any of these genes were essential for venetoclax sensitivity, their absence would lead to resistance. We introduced genetic perturbations by infecting Cas9-expressing OCI-AML2 and MOLM-13 AML cells with lentiviruses carrying a customized CRISPR knockout library. This library included 7 single guide RNAs (sgRNAs) per gene for the 1426 target genes, along with 250 sgRNAs designated as negative controls (Supplementary Table 1). Following transduction and antibiotic selection, cells were subjected to either venetoclax or vehicle (DMSO) treatment for 15 days. This timeframe allowed for approximately 10 cell doublings to ensure enrichment of resistance population. Genomic DNA was collected on the final day of treatment from both venetoclax and DMSO-treated cells. The PCR-amplified library of barcodes representing unique sgRNA sequences obtained from genomic DNA underwent deep sequencing and was analyzed using the MAGeCK pipeline (Fig. 1A). Our screen identified significant hits, with their depletion associated with changes in venetoclax sensitivity (Fig. 1B, C). Given that our CRISPR library was designed to target genes highly expressed in venetoclax-sensitive AML samples, we focused on the top hits, the depletion of which would lead to resistance to venetoclax. In the OCI-AML2 cell line, the loss of genes such as *CDK6*, *OGT*, *PDS5A*, *ZNF740*, *BCL2*, *TIPRL*, *NONO*, and *SMARCB1* were among the top enriched hits (Fig. 1B). In the MOLM-13 cells, several genes emerged as resistance hits, including *GTPBP3*, *ZNF740*, *NUBPL*, *MRPS30*, *ZNRF1*, *PUS1*, *VIRMA-DT*, and *TMEM17* (Fig. 1C). Remarkably, *ZNF740* was the sole top enriched gene common to the screen results in both OCI-AML2 and MOLM-13 AML cell lines. All 7 sgRNAs targeting ZNF740 showed significant enrichment under venetoclax treatment compared to DMSO control (Fig. 1D), indicating a strong consistency across independent sgRNA perturbations of the *ZNF740* gene.

The expression of ZNF740 showed a strong negative correlation with venetoclax AUC (*r* = −0.52, *p* = 2.71e-26) in the BeatAML 2.0 dataset (Fig. 1E), highlighting the clinical significance of ZNF740 in venetoclax therapeutic response. Previous research has shown that certain subtypes within the French-American-British (FAB) classification, particularly AML-M4 and AML-M5, which represent a more monocytic differentiation status of AML, exhibit resistance to venetoclax treatment [7, 14, 17]. Interestingly, we found that ZNF740 expression levels in AML-M4 and AML-M5 subtypes were significantly lower compared to the other FAB subtypes (Fig. 1F), which was consistent with our findings that ZNF740 loss results in venetoclax resistance. Moreover, the correlation between ZNF740 expression level and venetoclax therapeutic response was not limited to monocytic differentiation AML but also extended to other FAB subtypes (Supplementary Fig. 1C, D), suggesting a broader role for ZNF740 in venetoclax resistance in AML. Among common AML mutations, ZNF740 was found to be expressed at lower levels in AML subtypes associated with poor venetoclax response, such as those with KRAS mutations [7], and at higher levels in subtypes with better venetoclax response, such as NPM1 mutations [18] (Supplementary Fig. 1E).

### ZNF740 is required for sustaining venetoclax sensitivity in AML

**Fig. 2:**
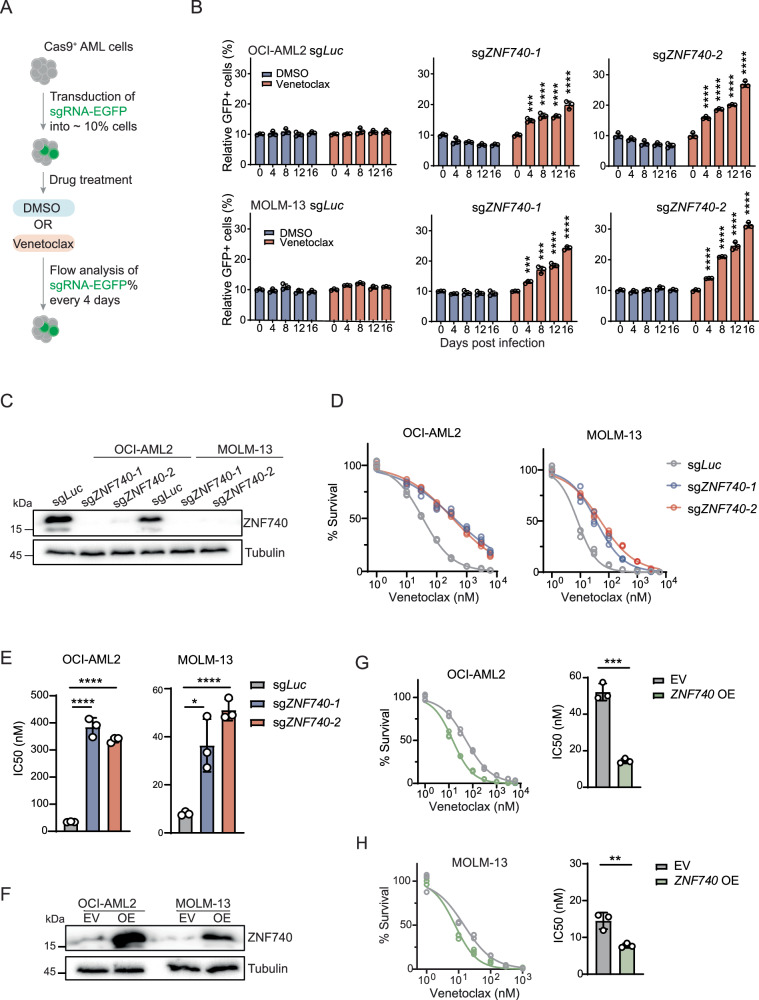
ZNF740 is required for sustaining venetoclax sensitivity in AML. **A** Schematic illustrating competitive growth assays to assess growth advantage of leukemia cells expressing specific sgRNA sequences, under conditions of either DMSO or venetoclax treatment. **B** Competitive proliferation assays showing the relative percentage of leukemia cells expressing EGFP-tagged sgRNA sequences under either DMSO or venetoclax treatment in OCI-AML2 (20 nM), and MOLM-13 (2 nM) cell lines. An sgRNA targeting the Luciferase gene sg*Luc* was used as a negative control. The EGFP percentage of each time point was normalized to the initial measurement. **C** Western blot analysis validating ZNF740 knockout in OCI-AML2 and MOLM-13 cell lines. **D** Dose-response curves showing viabilities of OCI-AML2 and MOLM-13 cell lines expressing indicated sgRNAs after a 4-day treatment with DMSO control or various doses of venetoclax. All cell viabilities were normalized to DMSO treatment. **E** Bar plots showing the calculated IC_50_ values from the dose-response curves. **F** Western blot analysis validating ZNF740 overexpression in OCI-AML2 and MOLM-13 cell lines. Dose-response curves showing viabilities of OCI-AML2 (**G**) and MOLM-13 (**H**) cell lines expressing either empty vector or exogenous ZNF740 after a 3-day treatment with DMSO control or various doses of venetoclax. All cell viabilities were normalized to DMSO treatment. Calculated IC_50_ values are shown in bar plots. *p < 0.05; **p < 0.01; ***p < 0.001; ****p < 0.0001.

To validate the role of ZNF740 in therapeutic response to venetoclax in AML cells, we utilized competitive proliferative assay to assess the impact of ZNF740 knockout on cell fitness following treatment with venetoclax or DMSO in OCI-AML2 and MOLM-13 cell lines (Fig. 2A). We selected two independent sgRNAs targeting ZNF740 and a luciferase-targeting guide RNA as a negative control. We confirmed that these two sgRNAs targeting ZNF740 significantly reduced the protein level of ZNF740 compared to control sgRNA using western blot analysis (Fig. 2C). Notably, cells carrying ZNF740 sgRNAs did not show affected proliferation in untreated conditions (Fig. 2B). However, when treated with venetoclax, cells harboring ZNF740 sgRNAs, in contrast to those with Luciferase sgRNA, exhibited a competitive advantage over non-infected cells (Fig. 2B), which is consistent with our screening results.

To explore the potential role of ZNF740 in venetoclax resistance, we established stable cell lines with ZNF740 wildtype and knockout in both OCI-AML2 and MOLM-13 cell lines. The depletion of ZNF740 in these cell lines resulted in significant resistance to venetoclax (Fig. 2D), with a much higher IC_50_ compared to those with wildtype ZNF740 (Fig. 2E). In addition, the absence of ZNF740 also conferred resistance to the combination of venetoclax and the hypomethylating agent 5-Azacytidine, a common regimen in AML treatment (Supplementary Fig. 2A, B). Since elevated ZNF740 expression correlates with increased sensitivity to venetoclax in primary AML samples (Fig. 1E), we examined if enhancing ZNF740 expression would further sensitize AML cells to venetoclax treatment. Interestingly, inducing ZNF740 overexpression in OCI-AML2 and MOLM-13 cell lines significantly increased their sensitivity to venetoclax treatment, in contrast to cells transfected with an empty vector (Fig. 2F–H). Together, these findings indicate that ZNF740 is both essential and adequate for enhanced venetoclax response in AML cells.

### ZNF740 loss diminishes apoptotic response to venetoclax in AML cells with increased MCL-1 protein expression

**Fig. 3:**
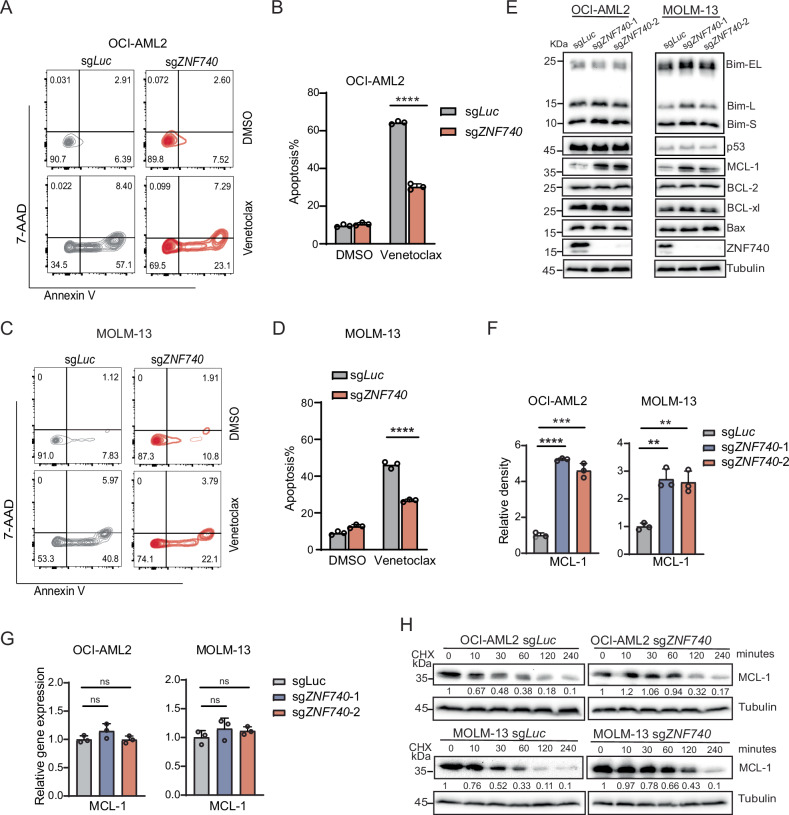
ZNF740 loss diminishes apoptotic response to venetoclax in AML cells with increased MCL-1 protein expression. **A**–**D** Flow cytometry analysis of the percentages of Annexin V and 7-AAD positive cells in OCI-AML2 (**A**) and MOLM-13 (**C**) cells expressing sg*Luc* and sg*ZNF740*, following a 3-day treatment with DMSO or venetoclax (OCI-AML2, 100 nM; MOLM-13, 10 nM). Bar plot depicting the percentages of Annexin V positive cells in the flow cytometry analysis of OCI-AML2 (**B**) and MOLM-13 (**D**) cells. **E** Western blot analysis showing the expression level indicated proteins following ZNF740 knockout in OCI-AML2 and MOLM-13 cell lines. **F** Bar plots depicting the relative density of MCL-1 western blot signals following ZNF740 knockout in OCI-AML2 and MOLM-13 cell lines. **G** RT-qPCR results showing the relative MCL-1 mRNA levels following ZNF740 knockout in OCI-AML2 and MOLM-13 cell lines. **H** Western blot analysis depicting MCL-1 protein stability after various times of treatment using cycloheximide (CHX, 50 µg/mL) in OCI-AML2 and MOLM-13 cell lines expressing sg*Luc* or sg*ZNF740*. Relative MCL-1 protein levels were indicated in numbers. **p < 0.01; ***p < 0.001; ****p < 0.0001; ns, not significant.

To evaluate the effect of ZNF740 knockout on venetoclax-induced cell death, we measured early and late apoptosis by quantifying Annexin-V and 7-AAD or DAPI positive cells through flow cytometry after venetoclax treatment. In both OCI-AML2 and MOLM-13 cell lines, cells with ZNF740 knockout showed a marked decrease in the percentage of apoptotic cells (Fig. 3A–D and Supplementary Fig. 3A, B).

The resistance mechanisms of venetoclax are closely linked to the dysregulation of anti-apoptotic proteins like BCL-2, MCL-1, BCL-xL, BCL2A1, and pro-apoptotic proteins, including BH3-only proteins (BAD, BIM, PUMA) and effector proteins (BAX, BAK), as well as mutations in TP53 [3, 6, 9, 19]. We next examined the expression levels of BCL-2, MCL-1, BCL-xL, BIM, BAX, and TP53 in OCI-AML2 and MOLM-13 cell lines following ZNF740 knockout using western blotting. Both cell lines demonstrated a significant reduction in ZNF740 protein. Interestingly, the expression levels of BCL-2, BCL-xL, BIM, BAX, and TP53 remained largely unchanged, indicating that the resistance induced by ZNF740 loss is not dependent on these proteins (Fig. 3E). However, we observed a notable and consistent increase in MCL-1 protein levels after ZNF740 knockout in OCI-AML2 and MOLM-13 cell lines (Fig. 3E, F). Elevated expression of MCL-1 is one of the important mechanisms of venetoclax resistance, and MCL-1 has been a promising target to overcome venetoclax resistance with multiple inhibitors being evaluated in clinical trials [20–22]. Considering ZNF740’s role in transcriptional regulation, we examined MCL-1 regulation at the mRNA level. Surprisingly, MCL-1 mRNA levels remained largely unchanged in ZNF740 knockout OCI-AML2 and MOLM-13 cell lines (Fig. 3G), suggesting that MCL-1 protein may be upregulated through post-transcriptional mechanisms. We then used cycloheximide (CHX) to inhibit protein synthesis in these cell lines and monitored MCL-1 protein stability. Notably, MCL-1 protein persisted longer in ZNF740 knockout cells post-CHX treatment, indicating enhanced stability compared to wildtype controls (Fig. 3H). Conversely, ZNF740 overexpression decreased MCL-1 protein stability (Supplementary Fig. 3C, D). In summary, we show that ZNF740 knockout leads to reduced apoptosis in response to venetoclax, which may primarily be due to increased MCL-1 protein stability.

### NOXA is a transcriptional target of ZNF740

**Fig. 4:**
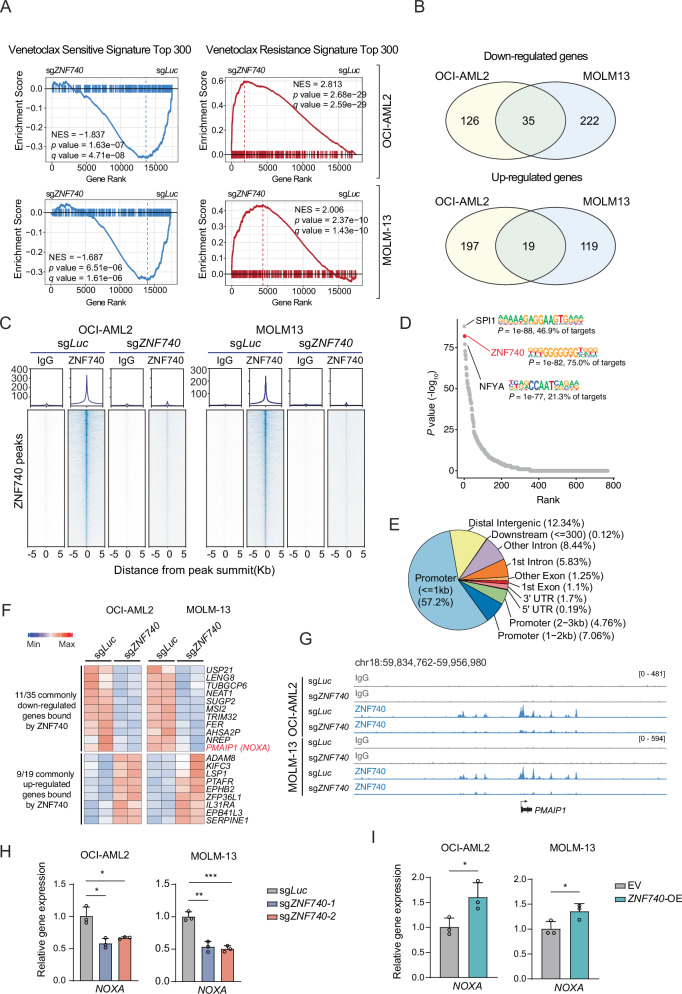
NOXA is a transcriptional target of ZNF740. **A** GSEA analysis revealing the impact of ZNF740 knockout on venetoclax-sensitive and -resistant signature genes in indicated cell lines. **B** Venn diagrams showing the commonly down- and up-regulated genes in OCI-AML2 and MOLM-13 cells following ZNF740 knockout. **C** Heatmap displaying the ZNF740 or IgG densities in ZNF740 peaks in indicated AML cells. **D** Homer motif analysis showing top enriched motifs in ZNF740 peaks. **E** Pie chart showing distribution of genomic localization of ZNF740 peaks. **F** Heatmaps showing expression of genes bound and commonly regulated by ZNF740 in OCI-AML2 and MOLM-13 cells. **G** CUT&RUN profiles at the NOXA locus in indicated leukemic cells. **H** RT-qPCR showing expression of NOXA in indicated AML cells with or without ZNF740 knockout. **I** RT-qPCR showing expression of NOXA in indicated AML cells with or without ZNF740 overexpression. *p < 0.05; **p < 0.01; ***p < 0.001.

To elucidate the role of ZNF740 in regulating the transcriptional landscape of AML cells, we conducted RNA sequencing (RNA-seq) on ZNF740 wildtype and knockout stable cell lines in OCI-AML2 and MOLM-13. This revealed 216 upregulated and 151 downregulated genes in OCI-AML2, and 138 upregulated and 257 downregulated genes in MOLM-13, following ZNF740 knockout (Supplementary Fig. 4A, B). Gene Set Enrichment Analysis (GSEA) of gene ontology biological processes identified that ZNF740 depletion led to increased expression of genes regulating the reactive oxygen species metabolism process (Supplementary Fig. 4C), which has been implicated in affecting the response to BCL2 inhibitors [23]. The differentiation state of AML influences its sensitivity to venetoclax treatment, with monocytic AML generally showing resistance to BCL2 inhibition [14, 17]. Our analysis of AML differentiation gene signatures [24] revealed that ZNF740 loss shifted cells from a primitive to a more monocytic AML state (Supplementary Fig. 4D). This shift was accompanied by a significantly increased expression of monocyte differentiation genes in ZNF740 knockout cells (Supplementary Fig. 4E, F). Finally, ZNF740 depletion resulted in a decreased expression of venetoclax-sensitive genes and increased expression of venetoclax-resistant genes (Fig. 4A), indicating that ZNF740 globally regulates genes linked to venetoclax sensitivity in AML.

Comparative RNA-seq analysis between OCI-AML2 and MOLM-13 cells identified 35 commonly down-regulated and 19 commonly up-regulated genes (Fig. 4B). To further narrow down the direct targets of ZNF740, we utilized CUT&RUN-sequencing with a ZNF740-specific antibody to map its genome-wide binding sites in both ZNF740 wildtype and knockout lines. The binding profiles of ZNF740 showed significant overlap between OCI-AML2 and MOLM-13 cells, revealing 12,982 common peaks (Supplementary Fig. 4G). Importantly, ZNF740 binding was eliminated after sgRNA-mediated gene knockout, confirming the specificity of the CUT&RUN assay (Fig. 4C). In agreement with this notion, the ZNF740 binding motif emerged as one of the most significantly enriched motifs (Fig. 4D). Genome-wide analysis of ZNF740-binding peaks indicated that about 70% localized to promoter regions (Fig. 4E), highlighting promoters as ZNF740’s primary action sites. Moreover, ZNF740 was also found binding to intergenic regulatory regions (Fig. 4E).

To identify the direct targets of ZNF740 in mediating venetoclax response, we integrated RNA-seq and CUT&RUN data and identified 11 commonly downregulated genes and 9 commonly upregulated genes that were directly bound by ZNF740 (Fig. 4F). Importantly, PMAIP1 (also known as NOXA) was identified as a direct target positively regulated by ZNF740. NOXA is a member of the BH3-only protein family and is known to play a role in enhancing venetoclax sensitivity and destabilizing MCL-1[25–27]. CUT&RUN analysis demonstrated ZNF740 binding at the promoter and multiple enhancer regions of the NOXA gene (Fig. 4G). Additionally, chromosome conformation capture data from 25 AML patients [28] indicated strong interactions between several distal enhancers and the NOXA promoter (Supplementary Fig. 4H). RT-qPCR validation confirmed that ZNF740 depletion decreased NOXA expression, whereas enforced ZNF740 overexpression elevated NOXA expression (Fig. 4H, I). These findings collectively establish NOXA as a transcriptional target of ZNF740.

### NOXA overexpression re-sensitizes venetoclax-resistant AML cells induced by ZNF740 depletion

**Fig. 5:**
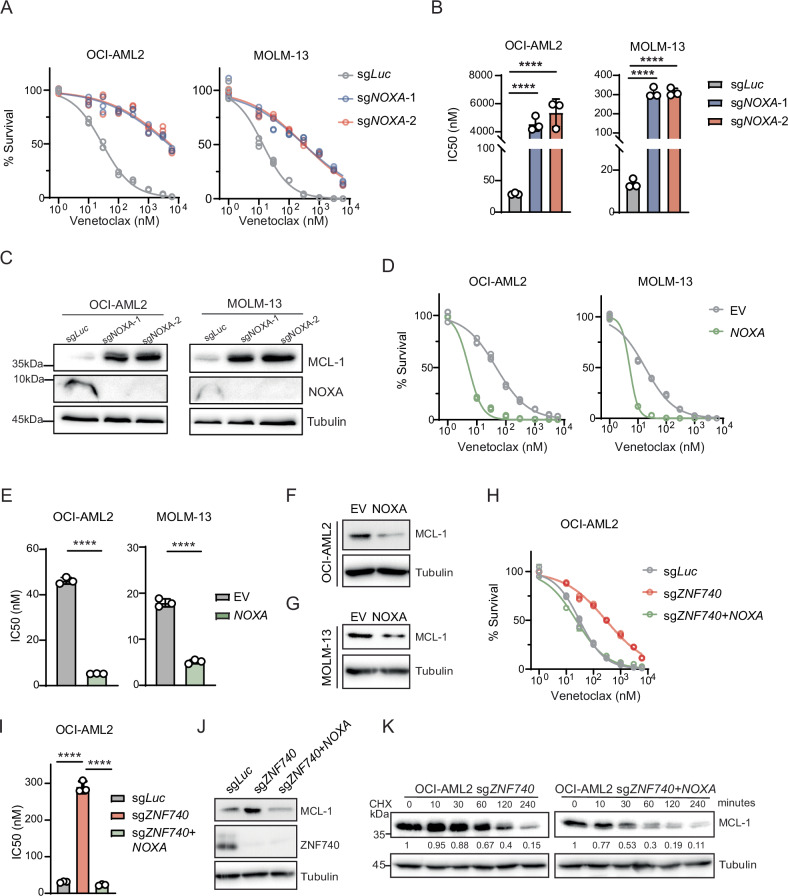
NOXA Overexpression Re-sensitizes Venetoclax-Resistant AML Cells Induced by ZNF740 Depletion. **A**, **B** Dose-response curves showing viability of OCI-AML2 and MOLM-13 cell lines expressing indicated sgRNAs after a 4-day treatment with DMSO control or various doses of venetoclax (**A**). All cell viabilities were normalized to DMSO treatment. Calculated IC_50_ values were shown in bar plots (**B**). **C** Western blot analysis showing MCL-1 protein expression in OCI-AML2 and MOLM-13 cell lines following by NOXA knockout. **D**, **E** Dose-response curves showing viability of OCI-AML2 and MOLM-13 cell lines expressing empty vector or exogenous NOXA after a 3-day treatment with DMSO control or various doses of venetoclax (**D**). All cell viabilities were normalized to DMSO treatment. Calculated IC_50_ values are shown in bar plots (**E**). Western blot analysis showing reduced MCL-1 protein levels followed by NOXA overexpression in OCI-AML2 (**F**) and MOLM-13 (**G**) cell lines. **H**, **I** Dose-response curves showing viability of OCI-AML2 cells expressing sg*Luc*, sg*ZNF740* alone or sg*ZNF740* combined with NOXA overexpression after a 4-day treatment with DMSO control or various doses of venetoclax (**H**). All cell viabilities were normalized to DMSO treatment. Calculated IC_50_ values are shown in bar plots (**I**). **J** Western blot analysis showing MCL-1 protein levels in indicated OCI-AML2 cells. **K** Western blot analysis depicting MCL-1 protein stability after various time of treatment using cycloheximide (CHX, 50 µg/mL) in cells expressing sgZNF740 alone or combined with NOXA overexpression of OCI-AML2 cell line. Relative MCL-1 protein levels were indicated in numbers. ****p < 0.0001.

Next, we investigated whether NOXA inactivation could mimic venetoclax resistance observed in ZNF740-deficient AML cells. We knocked out NOXA protein expression in OCI-AML2 and MOLM-13 using two independent sgRNAs (Fig. 5C). Similar to the resistance observed in ZNF740 knockout cells, NOXA depletion significantly increased resistance to the treatment with either venetoclax alone or the venetoclax/5-Azacytidine combination (Fig. 5A, B, Supplementary Fig. 5A, B). In line with the established role of NOXA in promoting MCL-1 degradation [27], we observed a marked increase in MCL-1 protein levels following NOXA loss (Fig. 5C). Conversely, overexpression of NOXA led to enhanced venetoclax sensitivity and reduced MCL-1 protein levels (Fig. 5D–G, Supplementary Fig. 5C). Next, we investigated whether reintroducing NOXA expression in ZNF740 knockout cells could reverse the venetoclax resistance phenotype. Remarkably, reintroducing NOXA in ZNF740-deficient cells restored venetoclax sensitivity and normalized MCL-1 levels (Fig. 5H–J) and decreased MCL-1 stability (Fig. 5K). Finally, we show that ZNF740 knockout failed to promote venetoclax resistance in NOXA-deficient OCI-AML2 and MOLM-13 cells (Supplementary Fig. 5D, E). These results collectively demonstrate that NOXA acts as a downstream effector of ZNF740 and plays a critical role in mediating venetoclax sensitivity by regulating MCL-1 protein stability.

### Combined treatment of MCL-1 inhibitor and venetoclax overcomes venetoclax resistance of ZNF740-deficient AML cells in vitro and in vivo

**Fig. 6:**
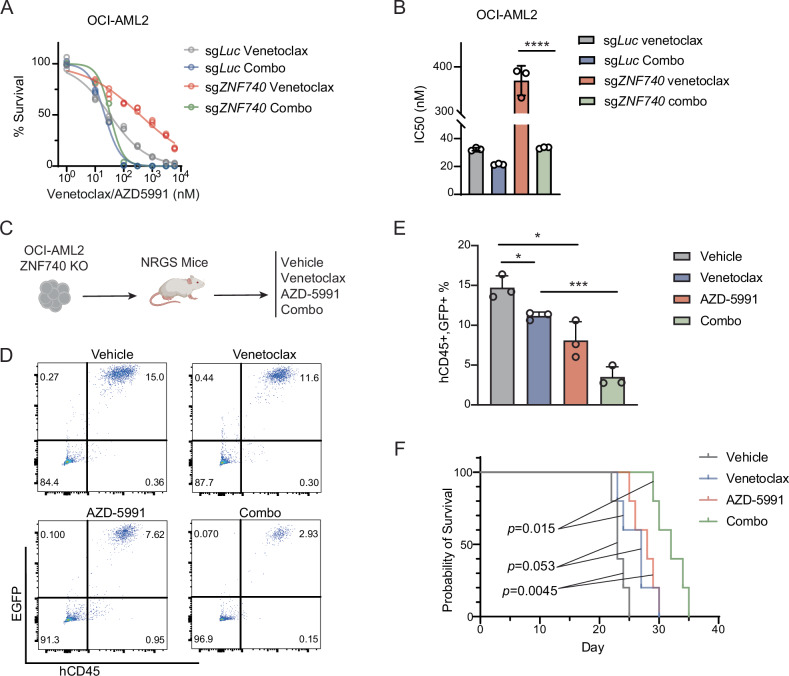
Combined treatment of MCL-1 inhibitor and venetoclax overcomes venetoclax resistance of ZNF740-deficient AML cells in vitro and in vivo. **A**, **B** Dose-response curves showing viabilities of OCI-AML2 expressing sg*Luc* or sg*ZNF740* after a 4-day treatment with various doses of venetoclax alone or combination treatment of venetoclax and AZD-5991 (**A**). All cell viabilities were normalized to DMSO treatment. Calculated IC_50_ values are shown in bar plots (**B**). **C** Schematic showing the transplantation of EGFP-expressing OCI-AML2 ZNF740 knockout cells into recipient mice, followed by a 3-week continuous treatment starting 5 days post-transplantation, using a vehicle control, venetoclax alone at 50 mg/kg daily, AZD-5991 alone at 100 mg/kg weekly, or combination of venetoclax and AZD-5991. **D**, **E** Flow cytometry analysis of the percentages of human CD45 and EGFP double-positive cells in bone marrow cells of the recipient mice 3 weeks post transplantation of indicated treatment groups. **F** Kaplan–Meier survival curves of mice after transplantation of OCI-AML2 ZNF740 knockout cells under indicated treatment. The *p* values were calculated by a log-rank test. *p < 0.05; ***p < 0.001; ****p < 0.0001.

Given the reported efficacy of several MCL-1 inhibitors in countering venetoclax resistance, we explored the potential of AZD-5991, an MCL-1 inhibitor with notable preclinical efficacy [29, 30], to mitigate venetoclax resistance in the context of ZNF740 loss. We treated wildtype and ZNF740 knockout OCI-AML2 cell lines with venetoclax, both alone and in combination with AZD-5991. The combination therapy significantly outperformed venetoclax monotherapy in reducing the IC_50_ in both wildtype and ZNF740 knockout lines (Fig. 6A, B). Remarkably, in ZNF740 knockout cells resistant to venetoclax, the combination of venetoclax and AZD-5991 reduced the IC_50_ to levels comparable to those in wildtype cells treated with venetoclax alone (Fig. 6B). To assess the in vivo efficacy, we transplanted EGFP-expressing venetoclax-resistant ZNF740 knockout OCI-AML2 cells into immune-deficient mice. The mice were then randomly grouped and treated with either vehicle, venetoclax, AZD-5991, or a combination of venetoclax and AZD-5991 (Fig. 6C). Single-agent treatment of either venetoclax or AZD-5991 demonstrated suboptimal effectiveness, as indicated by a modest decrease in hCD45 and EGFP double-positive AML cells in the bone marrow and an extension in survival compared to the vehicle group. However, the combination therapy markedly diminished AML cell engraftment in the bone marrow and significantly extended survival (Fig. 6D-F). These results suggest that pharmacological dual inhibition of MCL-1 and BCL-2 can effectively overcome venetoclax resistance associated with reduced ZNF740 expression.

## Discussion

The persistence of non-responsiveness or relapse after initial response to venetoclax remains a significant clinical hurdle. Deciphering the molecular mechanisms underlying venetoclax response is crucial for advancing treatment strategies. Gene expression profiling in primary AML samples has identified numerous genes associated with venetoclax response, as exemplified in BeatAML2.0 studies [16]. While these genes may serve as potential biomarkers for therapeutic outcomes, exploring their functional impact on venetoclax response could reveal new molecular regulators of drug response. In this study, by employing a comprehensive CRISPR screen approach, we discover that transcription factor ZNF740 is not only correlated with venetoclax responsiveness but also plays a key role in mediating the drug’s sensitivity. We further demonstrate that a deficiency in ZNF740 expression results in increased resistance to venetoclax, whereas enforced ZNF740 expression renders cells more sensitive to the drug. Our genome-wide profiling of ZNF740 targets and genetic rescue experiments further reveal the pro-apoptotic gene *NOXA* as a target of ZNF740. The loss of ZNF740 results in decreased NOXA expression, which in turn stabilizes MCL-1, a BCL-2-independent anti-apoptotic protein. This stabilization contributes to resistance against BCL-2 inhibition. Furthermore, we demonstrate that combined MCL-1 and BCL-2 inhibition is an effective strategy for treating AML cells with diminished ZNF740 expression.

Numerous venetoclax resistance mechanisms, such as genetic alterations, mitochondrial adaptations, mitophagy deregulation, lineage bias, and metabolic pathways, have been identified [5–15, 31]. Despite these advances, the regulatory mechanisms at the transcription level in this process remain largely unexplored. Here, we show that the zinc finger transcription factor ZNF740 is a previously uncharacterized regulator of venetoclax response. ZNF740 is a less-studied transcription factor with one reported role in regulating gene expression in heart [32]. In this study, we identify ZNF740 as a transcription factor mediating the anti-leukemic effects of BCL-2 inhibitors. We demonstrate that ZNF740 functions as an upstream activator of the pro-apoptotic gene NOXA in AML cell models, which subsequently downregulates MCL-1 protein levels. However, ZNF740 expression does not correlate with NOXA expression in the Beat AML dataset (data not shown), suggesting that ZNF740 may influence venetoclax response through mechanisms beyond NOXA modulation. Consistent with this notion, our findings show that ZNF740 knockout influences a spectrum of genes associated with venetoclax resistance and sensitivity, such as the reactive oxygen species metabolism genes and hematopoietic lineage signatures, which have been linked to regulating responses to BCL2 inhibition [14, 17, 23]. These results suggest that NOXA may not be the sole target of ZNF740 in regulating venetoclax response. For example, ZFP36L1, a gene bound by ZNF740 and upregulated following ZNF740 loss, has been implicated in promoting monocyte differentiation [33]. Further research is needed to explore the roles of these ZNF740 targets and their contributions to venetoclax response.

The BeatAML study, which evaluates drug responses through ex vivo testing of primary AML samples [16], typically addresses initial drug response rather than acquired resistance. Hence, our screen results suggest that ZNF740 may influence the up-front sensitivity to venetoclax, while its role in venetoclax resistance in relapsed AML post-treatment remains to be defined. In addition, it is important to correlate ZNF740 expression with clinical response in venetoclax-treated patients, which requires future studies with primary AML samples from both venetoclax responders and non-responders. Our current clinical data-focused CRISPR screening provides a framework for these subsequent functional studies to delineate the roles of genes differentially expressed between venetoclax responders and non-responders.

Together, our study comprehensively interrogates the functional roles of genes associated with clinical venetoclax sensitivity, which not only identifies ZNF740 as a key transcription factor regulating venetoclax sensitivity, but also implies potential strategies to improve venetoclax treatment response in AMLs with reduced ZNF740 expression.

## Methods

### Cell culture

AML cell lines OCI-AML2 and MOLM-13 were cultured in RPMI-1640 medium (Gibco) supplemented with 10% fetal bovine serum and 1% penicillin-streptomycin. Puromycin-resistant, Cas9-expressing MOLM-13, was kindly shared by Dr. Christopher R. Vakoc. Blasticidin-resistant, Cas9-expressing OCI-AML2 was generated by lentiviral transduction of lentiCas9-Blast (Addgene, 52962), followed by Blasticidin selection and validation of Cas9 expression and activity as previously described [34]. HEK293FT (Invitrogen) cells were cultured in DMEM (Gibco) supplemented with 10% fetal bovine serum and 1% penicillin-streptomycin. All cell lines were tested and confirmed to be free of mycoplasma contamination using MycoAlert PLUS Mycoplasma Detection Kit (Lonza).

### Plasmid cloning

Lentiviral vector LRG2.1_Neo (Addgene,125593) was used to express sgRNAs. For sgRNA cloning, vector was linearized with BsmBI (NEB) and ligated with specific sgRNAs using T4 DNA ligase (NEB). The sequences of sgRNAs used in this study are listed as follows: sg*Luc*,5’-CCC GGC GCC ATT CTA TCC GC-3’; sg*ZNF740*-1, 5’-GGA GCA GTT ACC ACC TAA AG-3’; sg*ZNF740*-2, 5’-GAG ACT TAC CAG TGT GAA TA-3’; sg*NOXA*-1, 5’-CGC TCAA CCG AGC CCC GCG C-3’; sg*NOXA*-2, 5’-TCG AGT GTG CTA CTC AAC TC-3’. For overexpression studies, human *ZNF740* (NM_001004304) and human *NOXA* (NM_021127) were cloned into a modified LeGO-iG2 (Addgene 27341) lentiviral vector by replacing EGFP with an EGFP-P2A-Neomycin cassette. All plasmids were verified by Sanger sequencing before use.

### Virus production and infection

Lentiviral particles were prepared using HEK293FT packaging cells (Invitrogen) via Nanofect (ALSTEM, NF100)-mediated transfection of gene delivery vector co-transfected with lentiviral packaging vectors psPAX2 and pMD2.G. Virus-containing supernatant was harvested 48 and 72 h after transfection and pooled. For infection, virus-containing supernatant was mixed with target cells supplied with 8 μg/mL polybrene (Sigma-Aldrich, TR-1003-G), and then centrifuged at 1500 rpm for 90 min at room temperature. Medium was replaced 24 h post-infection. Appropriate antibiotics were added 24 h post-infection when selection was needed. High infection efficiency was confirmed by flow cytometry or achieved by selection with antibiotics. Gene knockout or overexpression was confirmed by Western blotting for each experiment.

### Drug treatment

Venetoclax (HY-15531), 5-Azacytidine (HY-10586), AZD-5991 (HY-101533) were obtained from MedChemExpress. For in vitro drug treatment, leukemia cells were seeded in 24-well plates at a density of 20,000 cells/mL and treated with indicated dilutions of the inhibitors or 0.1% DMSO in culture medium (vehicle). Treated cells were re-plated into a new 24-well plate every 3 days with fresh medium and drug. Cell viability was measured at different time points using ATP-based luminescent viability assay CellTiter-Glo 2.0 (Promega), following the manufacturer’s instructions.

### Animal experiments

8–12-week-old NRGS mice (JAX, 024099) were given 2.5 Gy irradiation and then transplanted with 1 million cells via tail vein injection. Mice were randomly assigned to treatment groups and treated with vehicle (10% DMSO and 20% SBE-β-Cyclodextrin), 50 mg/kg venetoclax (formulated in 20% SBE-β-Cyclodextrin) daily by oral gavage, 100 mg/kg AZD-5991 (formulated in 30% 2-Hydroxypropyl-β-Cyclodextrin at pH 9.0) weekly through tail vein injection, or combination of both venetoclax and AZD-5991 starting 5 days post-transplant. Treatments were given for 3 weeks. Engraftment was monitored by assessing the hCD45 and EGFP double-positive cells in the bone marrow using flow cytometry. Sample sizes were determined by power analysis and an equal number of male and female mice were used in this study. Investigators were not blinded to treatment groups.

### Immunoblotting

Whole-cell extracts were prepared as previously described [35, 36], and subsequently separated by SDS-PAGE. Relative density of immunoblotting bands was analyzed using ImageJ. The following primary antibodies were used: anti-Tubulin (Proteintech, 66031-1-Ig), anti-ZNF740 (Proteintech, 25411-1-AP), anti-MCL-1 (Cell Signaling Technology, 5453S), anti-BCL-2 (Cell Signaling Technology, 15071S), anti-BCL-XL (Cell Signaling Technology, 2764S), anti-BAX (Cell Signaling Technology, 2772S), anti-BIM (Cell Signaling Technology, 2933S), anti-P53 (Santa Cruz, sc-126), and anti-NOXA (Abcam, ab13654).

### RT-qPCR

RNA was extracted from cells using the RNeasy kit (Qiagen, Germany) following the manufacturer’s instructions. First-strand cDNA was synthesized using the High-Capacity cDNA Reverse Transcription (RT) Kit (Applied Biosystems). Quantitative real-time PCR (qPCR) was performed in triplicate using the iTaq Universal SYBR Green Supermix (BioRad) on the CFX Opus 384 Real-Time PCR System (Bio-Rad). Expression levels were determined using the ΔΔCt method normalized to the housekeeping gene GAPDH. The primers used in this study were as follows: GAPDH forward, 5’- CCC ACC ACA CTG AAT CTC CC -3’; GAPDH reverse, 5’- TAC ATG ACA AGG TGC GGC TC -3’; NOXA forward, 5’- ACT GTT CGT GTT CAG CTC GC-3’; NOXA reverse, 5’- GAG TAG CAC ACT CGA CTT CCA -3’; MCL-1 forward, 5’- AAG AGG CTG GGA TGG GTT TGT G-3’; MCL-1 reverse, 5’- TTG GTG GTG GTG GTG GTT GG-3’.

### Flow cytometry analysis

Leukemia cells were collected after treatment and analyzed on an LSR Fortessa (BD Biosciences) flow cytometer. Data were analyzed using FlowJo (BD Biosciences) software. Mouse bone marrow cells were harvested and stained with human CD45-APC antibodies (BioLegend, 368612). For apoptosis analysis, APC Annexin V apoptosis detection kit with either 7-AAD (BioLegend) or DAPI (BioLegend) was used following the manufacturer’s instructions.

### CRISPR screen and data analysis

The CRISPR knockout library included 1426 genes whose expression inversely correlates with venetoclax AUC (Pearson correlation *r* < −0.4) in the BeatAML 2.0 dataset [16]. Each gene was represented by 7 sgRNAs, and an additional 250 sgRNAs were included as negative controls. sgRNA oligos were synthesized by Genscript and cloned into the LRG2.1_Neo (Addgene, 125593) vector using Gibson Assembly. Lentiviruses of this CRISPR knockout library were packaged in HEK293T cells and were used to infect OCI-AML2 cells and MOLM-13 cells at less than 0.5 MOI to ensure that each cell contained no more than one sgRNA. The transduced cells were selected by Neomycin for 5 days (T0) and treated with either DMSO or venetoclax (50 nM for OCI-AML2 and 10 nM for MOLM-13) for 15 days. Cells with at least 1,000x coverage (i.e., 10 million cells) were maintained throughout the screen, treatment, and sample collection. Genomic DNA was isolated and sgRNA sequences were amplified by two round PCR reactions using Nextera primers as described before [37], followed by deep sequencing on the NovaSeq 6000 PE150 platform. After sequencing, sgRNA counts were extracted and mapped to sgRNA sequences in the library and analyzed using by MAGeCK [38].

### RNA-seq and data analysis

RNA-seq libraries were prepared using the NEBNext Ultra RNA Library Prep Kit for Illumina (New England Biolabs) as previously described [39]. The quality and concentration of the libraries were examined using the Bioanalyzer High Sensitivity DNA Chip (Agilent). The multiplexed RNA-seq libraries were paired-end sequenced for 150 bp on the Illumina HiSeq 6000 platform. The paired-end reads were mapped to the human genome (hg38) using STAR with default parameters. The read counts were used for DESeq2 to define differentially expressed genes. Gene set enrichment analysis (GSEA) was performed to evaluate the enrichment of venetoclax response signatures, defined as the top 300 highly expressed genes by comparing gene expression in samples with the top and bottom 10% of venetoclax AUCs in the BeatAML2.0 dataset.

### CUT&RUN

CUT&RUN assay was performed as previously described [40–42]. Cells were collected and washed with Wash Buffer (20 mM HEPES pH 7.5, 150 mM NaCl, 0.5 mM Spermidine, protease inhibitor cocktail). After washing, cells were resuspended with Wash Buffer and incubated with activated Concanavalin A beads (Bangs Laboratories). Cell-binding beads were resuspended with Antibody Buffer (Wash Buffer plus 0.01% Digitonin and 2 mM EDTA) and primary antibody then incubated at 4 °C overnight with rotation. On day 2, cell-binding beads were washed with ice-cold Digitonin Buffer (Wash Buffer plus 0.01% Digitonin) twice and incubated with 2.5 µl/sample pAG-MNase (EpiCypher, 15-1116). Targeted digestion was performed on ice with 1 µl/sample 100 mM CaCl_2_ for 2 h and stopped by adding 33 µl/sample of Stop Buffer (340 mM NaCl, 20 mM EDTA, 4 mM EGTA, 50 µg/mL RNase A, 50 µg/mL glycogen). DNA was purified using the DNA Clean & Concentrate-5 kit (Zymo Research, D4014) and proceeded to library preparation using the NEBNext Ultra II Library Prep kit (New England Biolabs). CUT&RUN libraries were sequenced on the NovaSeq 6000 PE150 platform. The following antibodies were used in CUT&RUN assays: anti-ZNF740 (Proteintech, 25411-1-AP), and anti-rabbit IgG (Cell Signaling Technology, 2729S).

### CUT&RUN data analysis

Raw CUT&RUN reads were subjected to adapter removal (cutadapt 2.10) and mapped genome hg38 (bowtie2 2.4.1) [43] using --end-to-end --very-sensitive -–no-mixed -–no-discordant --phred33 -I 10 -X 700 parameters. Bigwig files were generated by bamCoverage [44] (version 3.3.0) and used for visualization in IGV [45] (version 2.8.6). Heatmaps were generated by deeptools [44] (version 3.3.0). MACS2 (v2.2.7.0) were used for peak calling from CUT&RUN against IgG controls. HOMER [46] was used for de novo motif analysis using the HOCOMOCO human transcription factor database.

### Virtual 4C analysis

We obtained the 25 AML patients’ data in pairs format [47] and aggregated the contacts from all 25 AML patients. We conducted Virtual 4 C analysis as previously described [47]. In summary, we used the PMAIP1 (NOXA) promoter region (hg38, chr18:59,899,096-59,900,096) as the bait and extracted the rows overlapping with the bait and its flanking regions from the 1 kb bin Hi-C matrix to determine the contact numbers. Subsequently, we plotted the observed contact numbers with a smoothing window (250 bp) to generate Virtual 4C profiles.

### Statistics

Data are presented as the mean ± SD from three independent experiments unless otherwise noted. The sample size was determined by power analysis. No randomization method was used, and no data were excluded from analysis. Statistical analyses were performed by a two-tailed Student’s test for comparing two data sets with assumed normal distribution unless otherwise noted. A Fisher’s exact test was performed for categorical variables. Survival analyses were performed using a log-rank test with GraphPad Prism software (v9). *, **, ***, and **** denote *p* values < 0.05, 0.01, 0.001, and 0.0001, respectively. n.s. denotes not significant.

### Supplementary information

Supplemental information

Supplemental table

original data files

## Data Availability

The RNA-seq and CUT&RUN-seq datasets are available at the Gene Expression Omnibus (GEO) under the accession numbers GSE267342 and GSE267343, respectively.

## Code availability

The code used for the RNA-seq and Cut&Run data analysis can be obtained from the authors upon request by email.
