# Supplementary material for: CRISPR screen of venetoclax response-associated genes identifies transcription factor ZNF740 as a key functional regulator
Source: Cell Death Dis. 2024 Aug 27;15(8):627. doi: 10.1038/s41419-024-06995-x (PMC11350041; doi:10.1038/s41419-024-06995-x)
Supplement: Supplementary file 1 — Supplemental information [file 41419_2024_6995_MOESM1_ESM.pdf]

Supplementary Information

CRISPR Screen of Venetoclax Response-Associated Genes Identifies Transcription Factor ZNF740 as a Key Functional Regulator

Lixia Zhang, Xinyue Zhou, Sajesan Aryal, Virginia Veasey, Pengcheng Zhang, Fu Jun Li, Yu Luan, Ravi Bhatia, Yang Zhou, Rui Lu

Table of Contents

Supplemental Figures
Supplemental Figure 1
Supplemental Figure 2
Supplemental Figure 3
Supplemental Figure 4
Supplemental Figure 5

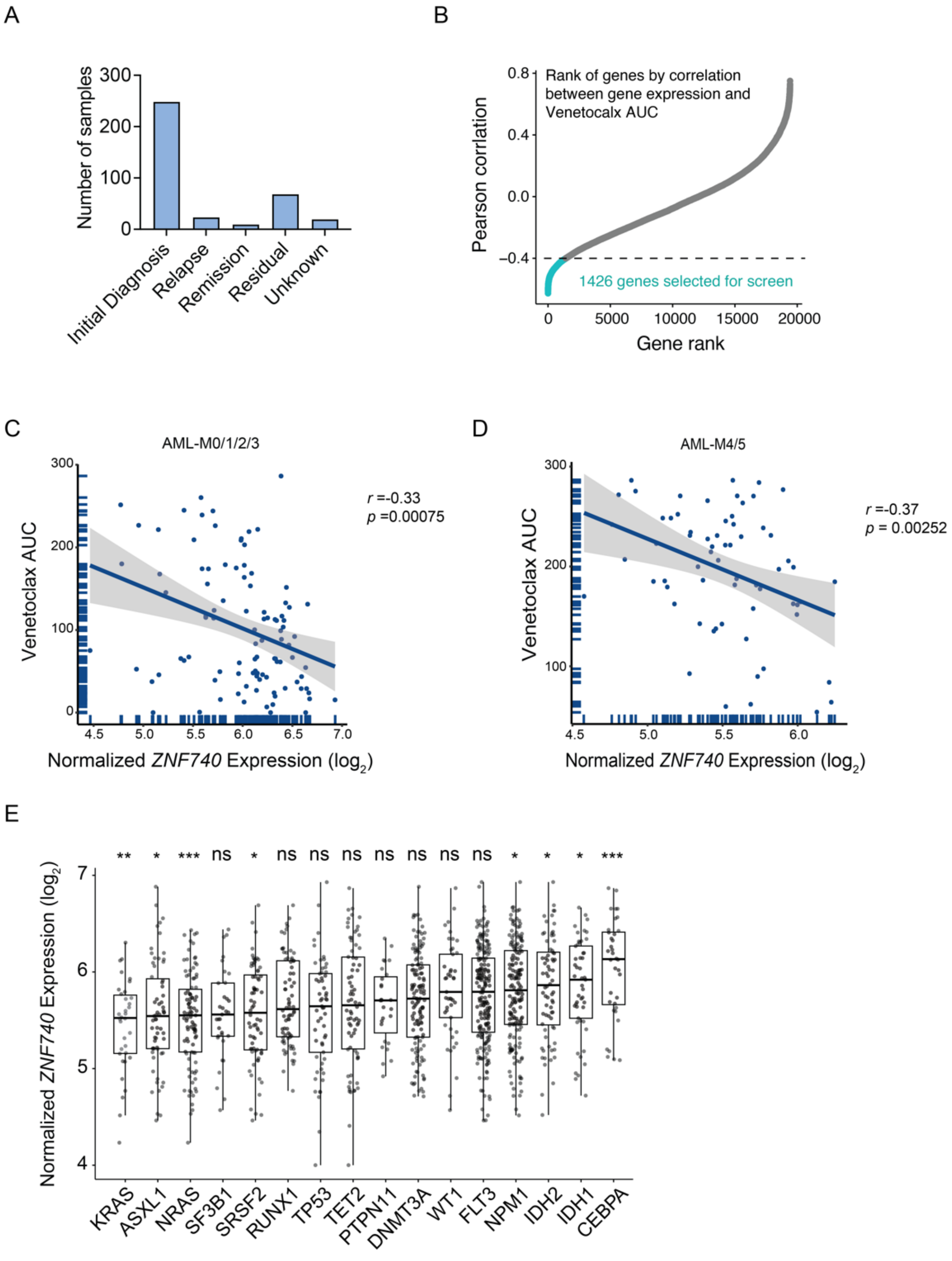
A
Number of samples
300
200
100
0
Initial Diagnosis
Relapse
Remission
Residual
Unknown
B
Pearson corrlation
0.8
0.4
0.0
−0.4
Rank of genes by correlation between gene expression and Venetocalx AUC
1426 genes selected for screen
0
5000
10000
15000
20000
Gene rank
C
AML-M0/1/2/3
Venetoclax AUC
300
200
100
0
r =-0.33
p =0.00075
4.5
5.0
5.5
6.0
6.5
7.0
Normalized ZNF740 Expression (log2)
D
AML-M4/5
Venetoclax AUC
300
200
100
r =-0.37
p = 0.00252
4.5
5.0
5.5
6.0
Normalized ZNF740 Expression (log2)
E
** * *** ns * ns ns ns ns ns ns ns * * * ***
Normalized ZNF740 Expression (log2)
7
6
5
4
KRAS
ASXL1
NRAS
SF3B1
SRSF2
RUNX1
TP53
TET2
PTPN11
DNMT3A
WT1
FLT3
NPM1
IDH2
IDH1
CEBPA

Supplemental Figure 1. Screen of Venetoclax Response-Associated Genes Reveals Transcription Factor ZNF740 as a Key Regulator.

A. Disease stage of BEAT AML samples used in this analysis.

B. Ranking of genes based on the correlation of their expression with venetoclax AUC. The top 1,426 genes demonstrating a correlation coefficient less than -0.4 were selected for the CRISPR screen.

C-D. Scatter plots representing the negative correlation between normalized ZNF740 expression levels and venetoclax AUCs in AML patient samples of AML-M0/1/2/3 (A) and AML-M4/5 (B) from the BeatAML2.0 dataset.

E. Bot plot representing normalized ZNF740 expression among common AML mutation groups. Significance was determined by comparing samples with each mutation to all other samples using two-tailed Mann–Whitney tests and corrected for multiple comparisons.

*, $p < 0.05$; **, $p < 0.01$; ***, $p < 0.001$; ns, not significant.

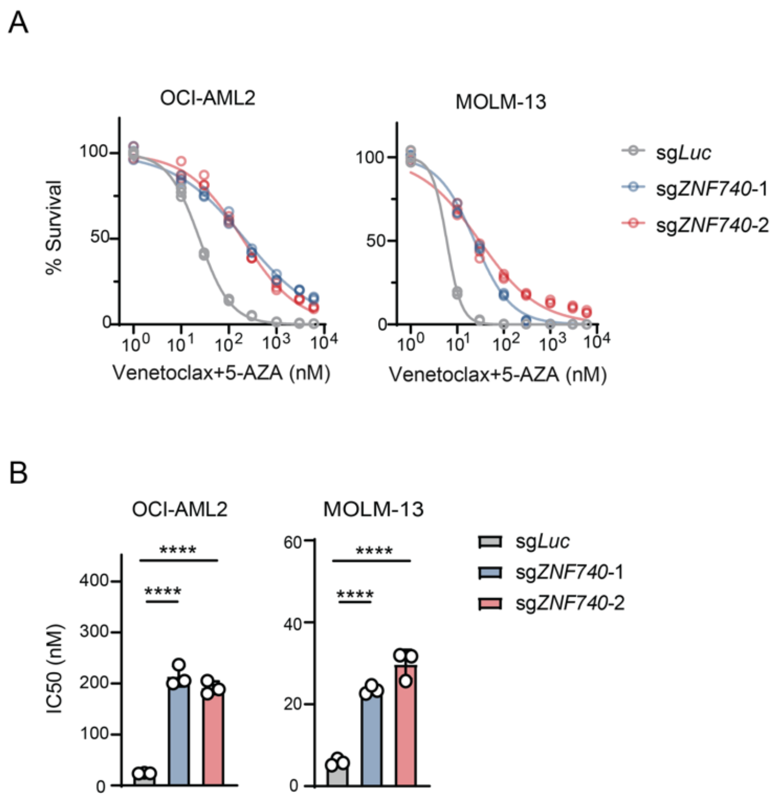

Supplemental Figure 2. ZNF740 is Required for Sustaining Venetoclax Sensitivity in AML.

A. Dose-response curves showing viabilities of OCI-AML2 and MOLM-13 cell lines expressing indicated sgRNAs after a 4-day treatment with DMSO control or various doses of venetoclax combined with 5-Azacytidine. All cell viabilities were normalized to DMSO treatment.

B. Bar plots showing the calculated IC_{50} values from the dose-response curves.

****, $p < 0.0001$.

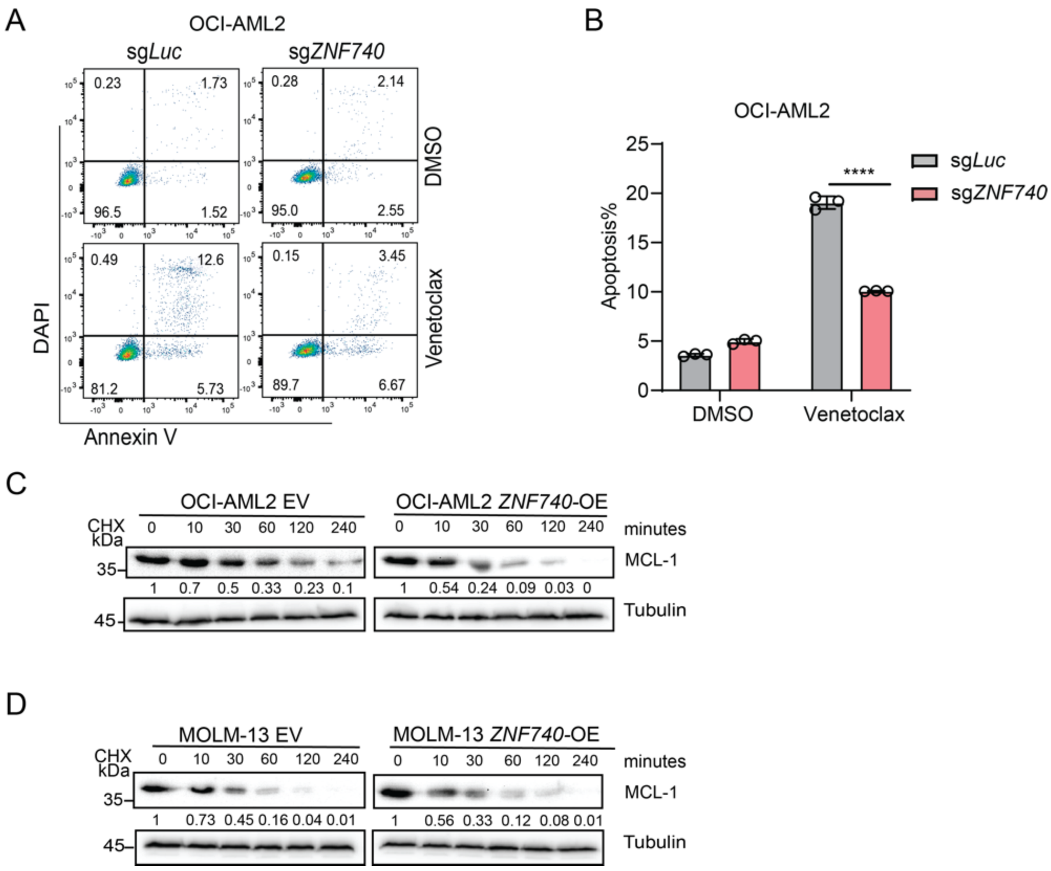

Supplemental Figure 3. ZNF740 Loss Diminishes Apoptotic Response to Venetoclax in AML Cells with Increased MCL-1 Protein Expression.

A. Flow cytometry analysis of the percentages of Annexin V and DAPI positive cells in OCI-AML2 cells expressing sg*Luc* and sg*ZNF740*, following a 48-hour treatment with DMSO or 100 nM venetoclax.

B. Bar plot depicting the percentages of Annexin V positive cells in the flow cytometry analysis of OCI-AML2 as shown in A.

C-D. Western blot analysis depicting MCL-1 protein stability after various time of treatment using cycloheximide (CHX, 50 μg/mL) in cells expressing empty vector or overexpressing ZNF740 of OCI-AML2 and MOLM-13 cell lines. Relative MCL-1 density was indicated in numbers.

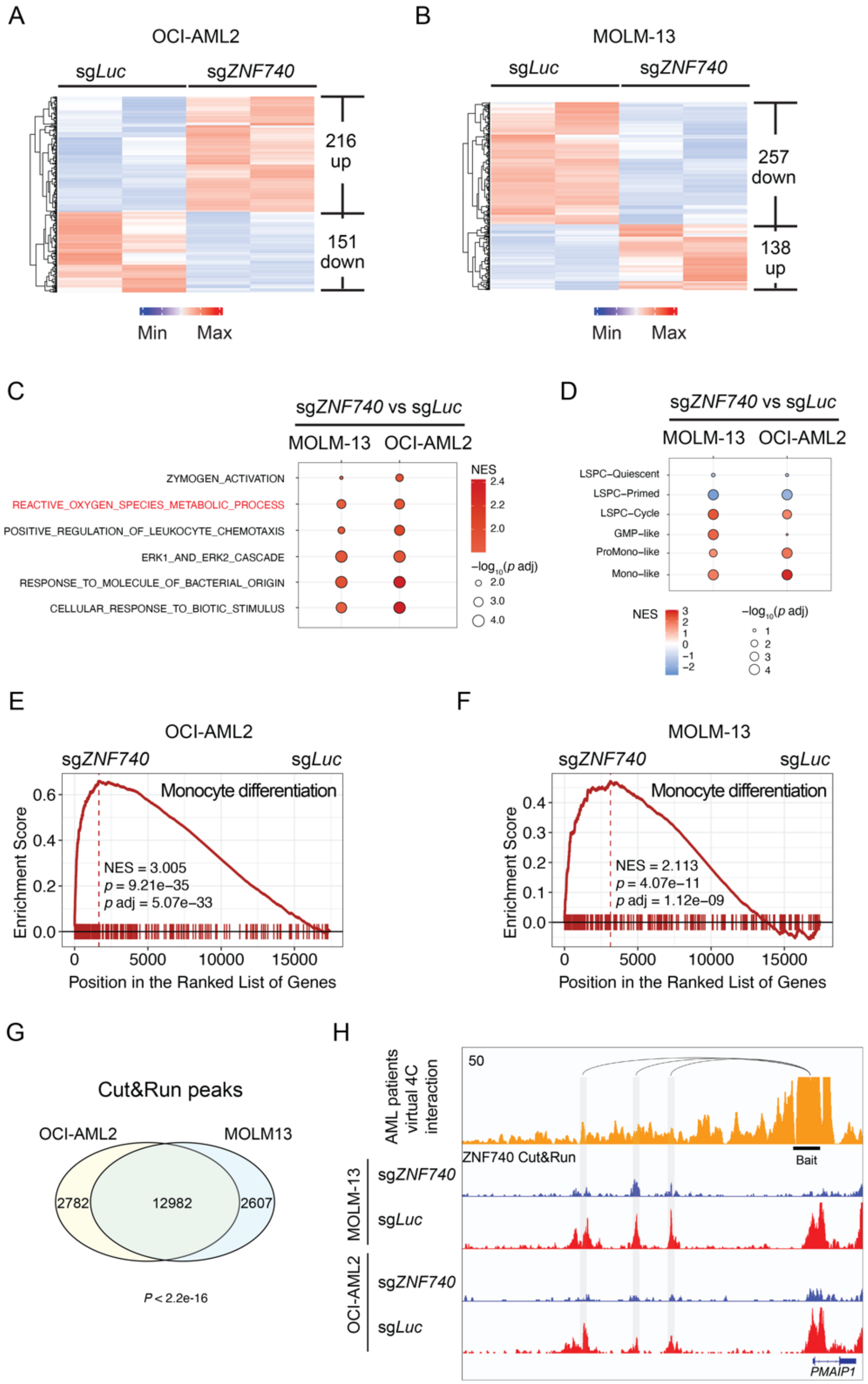
A
OCI-AML2
sgLuc
sgZNF740
216
up
151
down
Min
Max
B
MOLM-13
sgLuc
sgZNF740
257
down
138
up
Min
Max
C
sgZNF740 vs sgLuc
MOLM-13
OCI-AML2
ZYMOGEN_ACTIVATION
REACTIVE_OXYGEN_SPECIES_METABOLIC_PROCESS
POSITIVE_REGULATION_OF_LEUKOCYTE_CHEMOTAXIS
ERK1_AND_ERK2_CASCADE
RESPONSE_TO_MOLECULE_OF_BACTERIAL_ORIGIN
CELLULAR_RESPONSE_TO_BIOTIC_STIMULUS
NES
2.4
2.2
2.0
−log10(p adj)
2.0
3.0
4.0
D
sgZNF740 vs sgLuc
MOLM-13
OCI-AML2
LSPC−Quiescent
LSPC−Primed
LSPC−Cycle
GMP−like
ProMono−like
Mono−like
NES
3
2
1
0
−1
−2
−log10(p adj)
1
2
3
4
E
OCI-AML2
sgZNF740
sgLuc
Monocyte differentiation
NES = 3.005
p = 9.21e−35
p adj = 5.07e−33
Enrichment Score
Position in the Ranked List of Genes
F
MOLM-13
sgZNF740
sgLuc
Monocyte differentiation
NES = 2.113
p = 4.07e−11
p adj = 1.12e−09
Enrichment Score
Position in the Ranked List of Genes
G
Cut&Run peaks
OCI-AML2
MOLM13
2782
12982
2607
P < 2.2e-16
H
AML patients virtual 4C interaction
50
Bait
ZNF740 Cut&Run
MOLM-13
sgZNF740
sgLuc
OCI-AML2
sgZNF740
sgLuc
PMAIP1

Supplemental Figure 4. NOXA is a Transcriptional Target of ZNF740.

A-B. Heat map showing the transcriptional analysis of differentially expressed genes (fold change > 1.2 and p-value < 0.05) between ZNF740 wildtype and knockout cells in OCI-AML2 (A) and MOLM-13 (B) cell lines.

C. Bubble plot showing top enriched pathways from Gene Ontology Biological Processes (GO BP).

D. Bubble plot showing enrichment of genes specific to AML cell states.

E-F. GSEA showing increased monocyte differentiation signature genes in OCI-AML2 (E) and MOLM-13 (F) cells following ZNF740 knockout.

G. Venn diagram showing the overlap of ZNF740 peaks identified in OCI-AML2 and MOLM-13 cell lines.

H. Chromatin interactions between the ZNF740 bound distal enhancers and *NOXA* promoter (bait) as determined by virtual 4C analysis of AML HiC data.

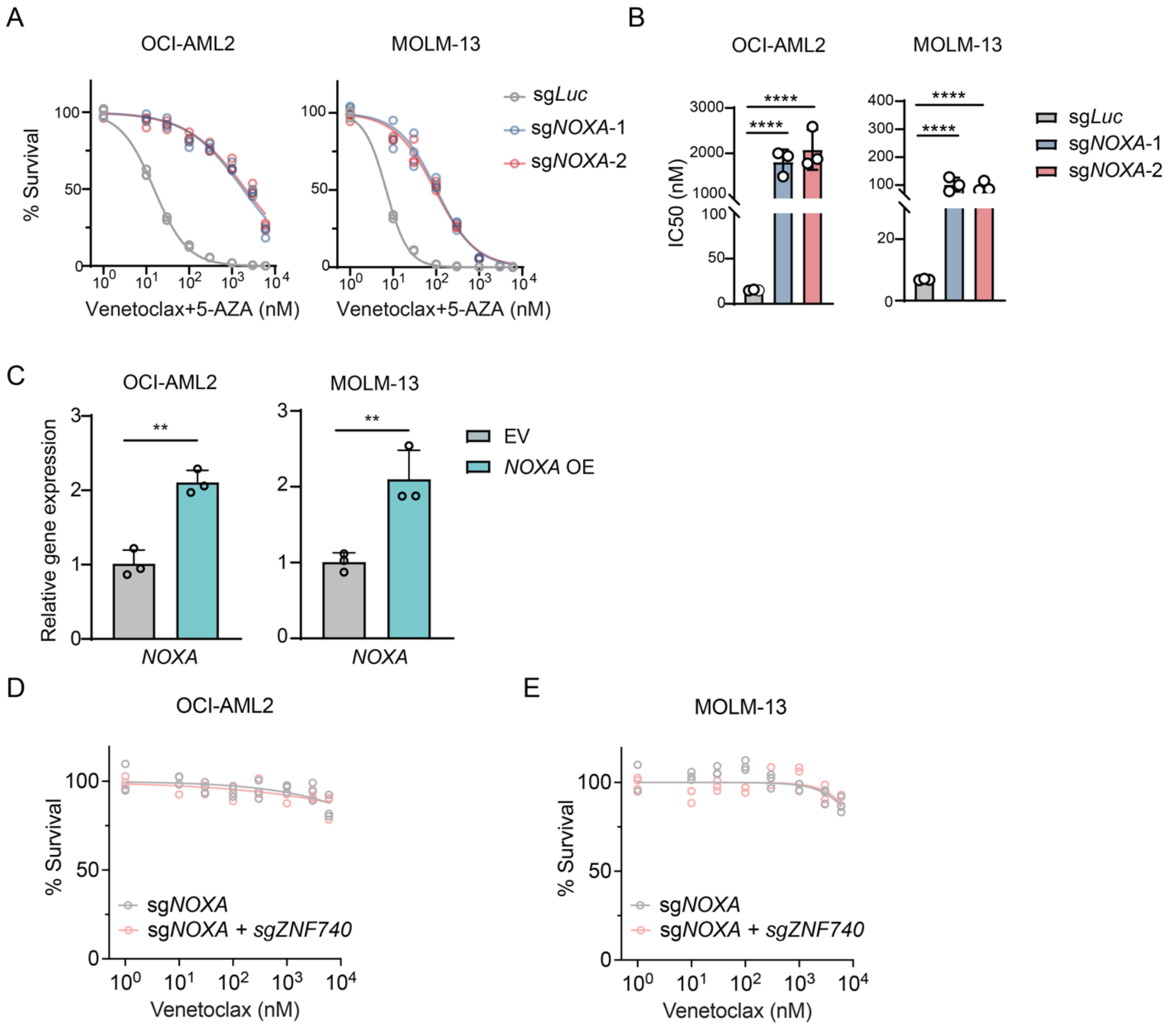

Supplemental Figure 5. NOXA Overexpression Re-sensitizes Venetoclax-Resistant AML Cells Induced by ZNF740 Depletion.

A. Dose-response curves showing the viability of OCI-AML2, and MOLM-13 cell lines expressing indicated sgRNAs after a 4-day treatment with DMSO control or various doses of venetoclax combined with 5-Azacytidine. All cell viabilities were normalized to DMSO treatment.

B. Bar plots showing the calculated IC_{50} values from the dose-response curves.

C. RT-qPCR depicting the overexpression of NOXA mRNA in OCI-AML2, and MOLM-13 cell lines.

D-E. Dose-response curves showing viability of indicated OCI-AML2 (D) and MOLM-13 (E) cell lines after a 3-day treatment with DMSO control or various doses of venetoclax. All cell viabilities were normalized to DMSO treatment.

**, $p < 0.01$; ****, $p < 0.0001$.
