# Supplementary material for: CRISPR screen of venetoclax response-associated genes identifies transcription factor ZNF740 as a key functional regulator
Source: Cell Death Dis. 2024 Aug 27;15(8):627. doi: 10.1038/s41419-024-06995-x (PMC11350041; doi:10.1038/s41419-024-06995-x)

Supplemental Materials (Figure 2)

Figure 2C

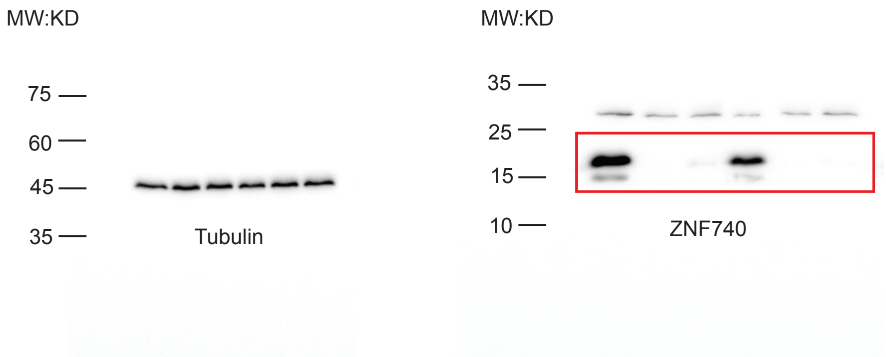

Figure 2F

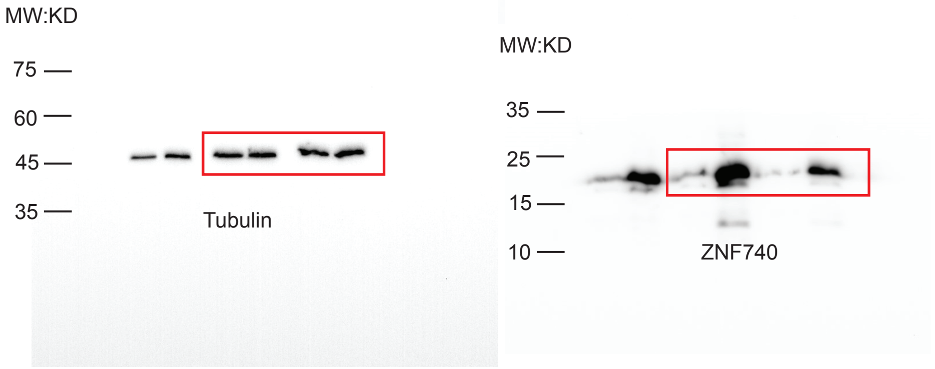

Supplemental Materials (Figure 3)

Figure 3E

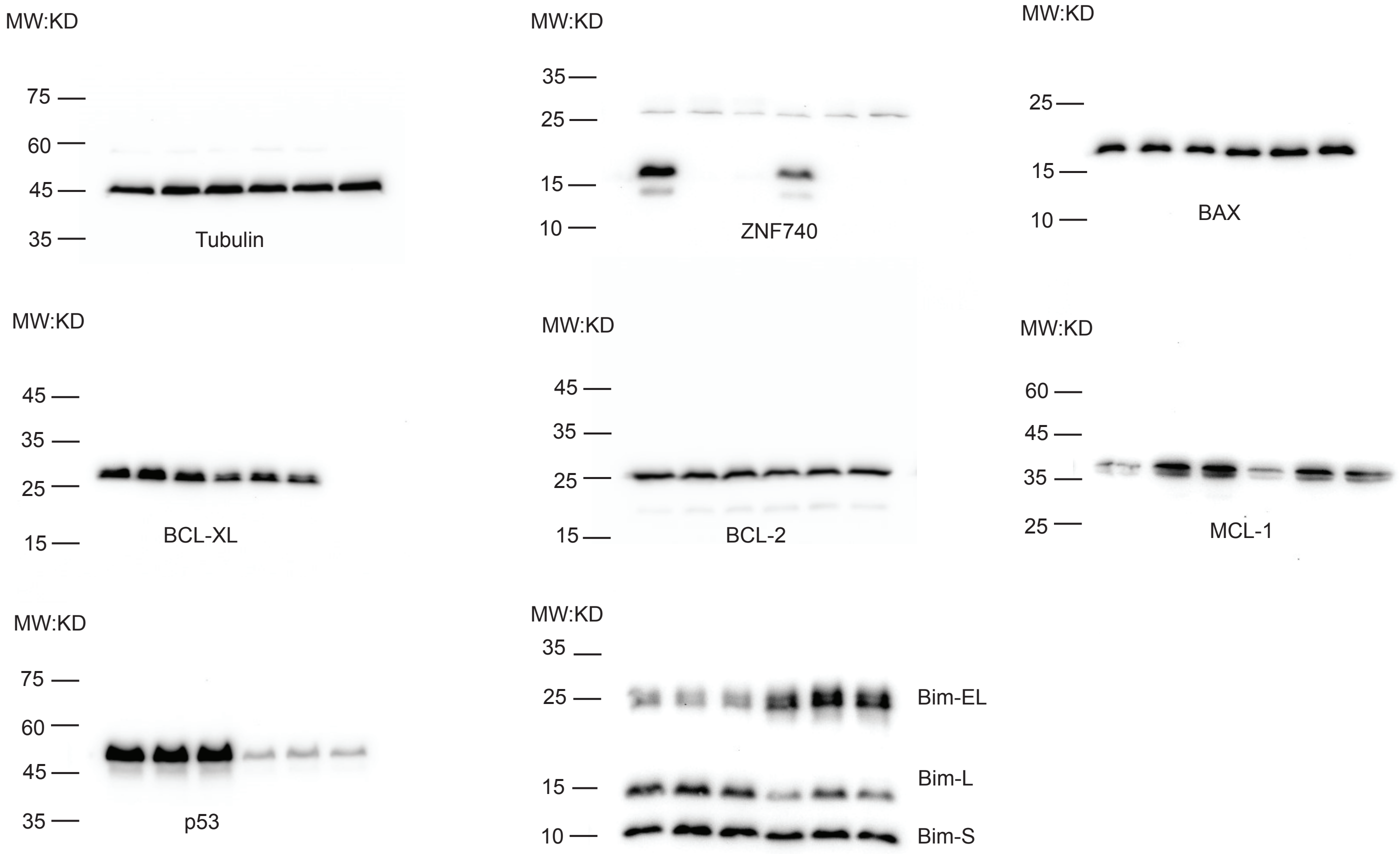

Figure 3H

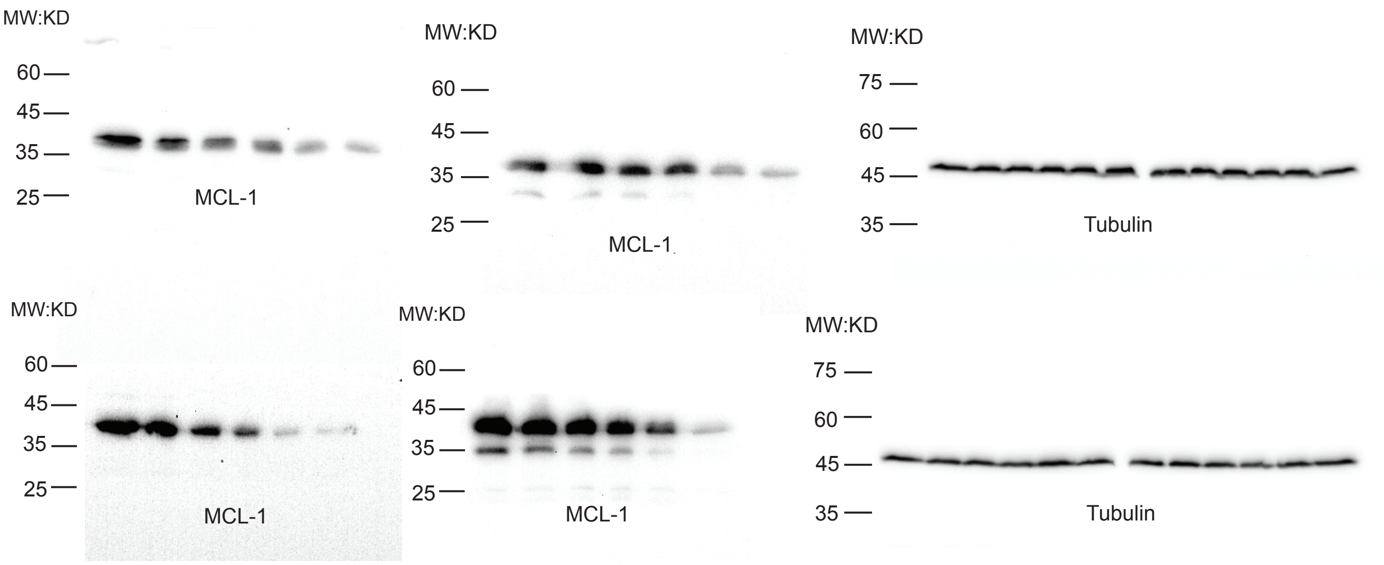

Supplemental Materials (Figure 5)

Figure 5C

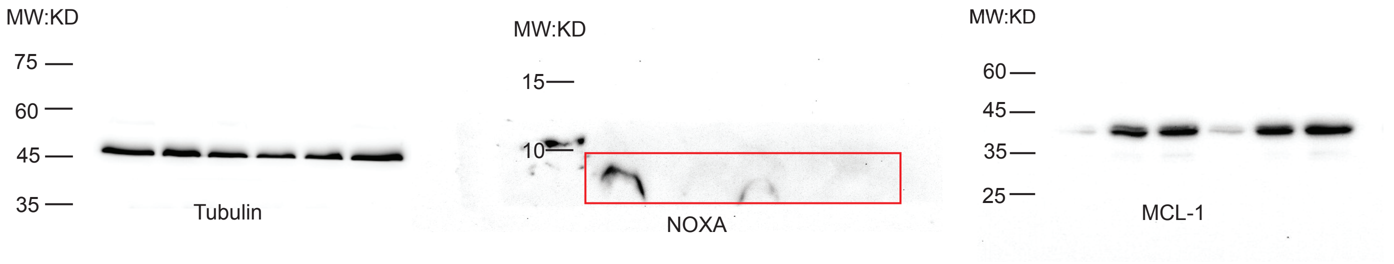

Figures 5F and 5J

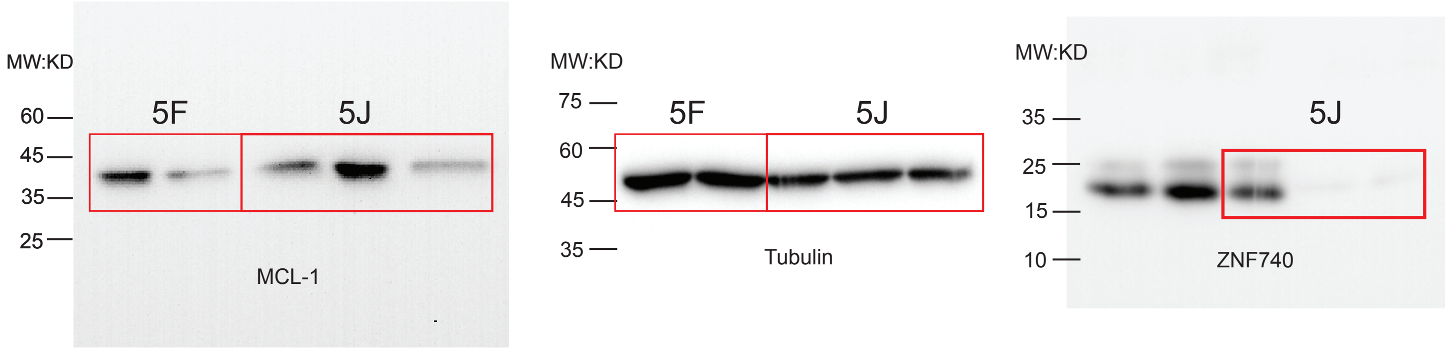

Figure 5G

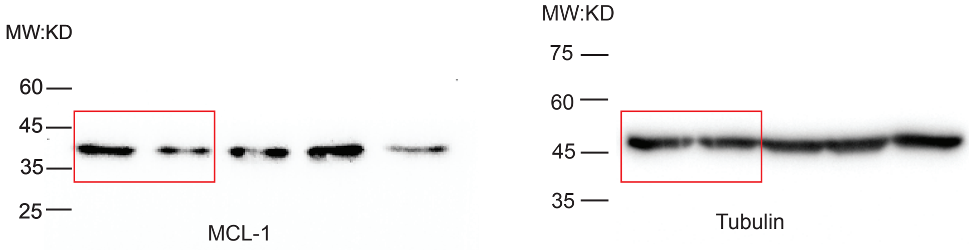

Figure 5K

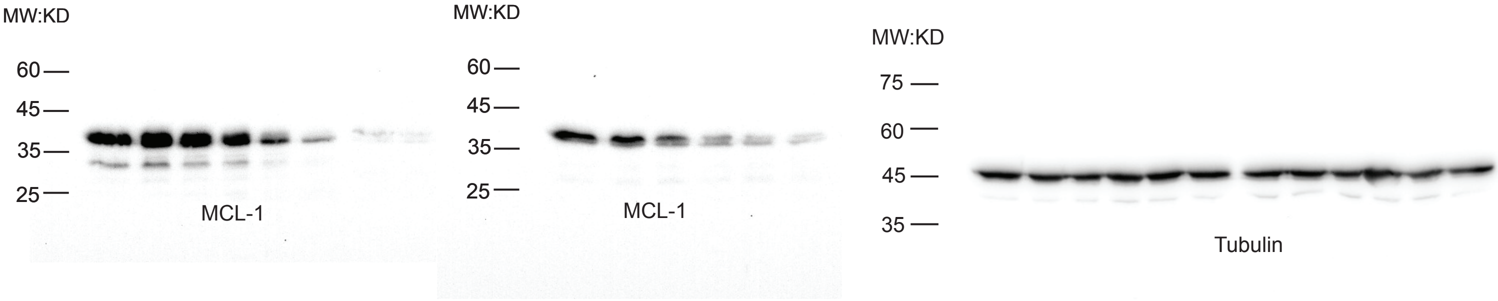

Supplemental Materials(Supplemetal Figure 3)

Figure S3A

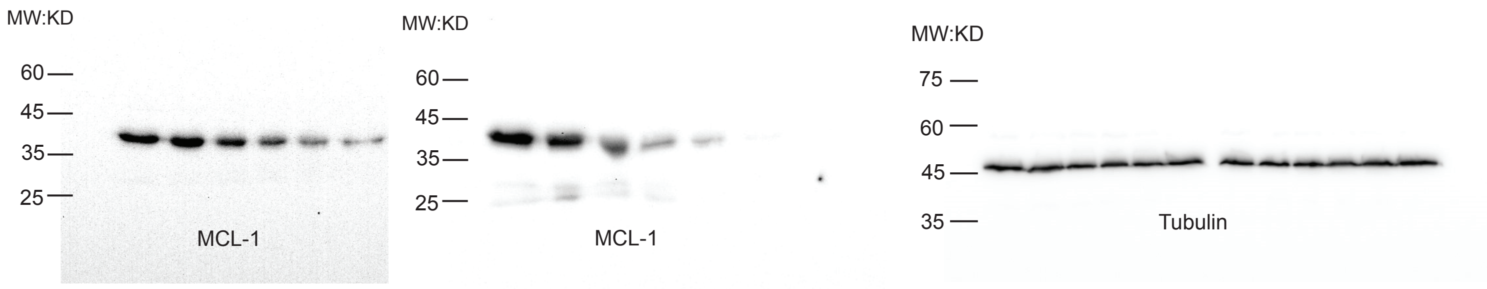

Figure S3B

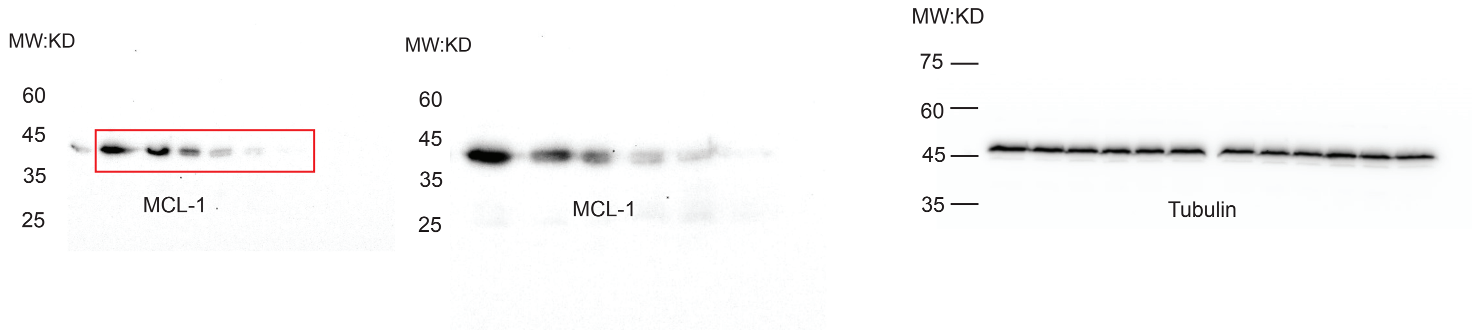

Supplement: Supplementary file 3 — original data files [file 41419_2024_6995_MOESM3_ESM.pdf]
